# Prothrombin complex concentrate restores haemostasis in a dabigatran anticoagulated polytrauma pig model

**DOI:** 10.1186/cc13305

**Published:** 2014-03-17

**Authors:** M Honickel, J Van Ryn, HM Spronk, H Ten Cate, R Rossaint, O Grottke

**Affiliations:** 1 RWTH Aachen University Hospital, Aachen, Germany; 2 CardioMetabolic Diseases Research, Boehringer Ingelheim GmbH & Co. KG, Biberach, Germany; 3 Maastricht University Medical Center, Maastricht, the Netherlands

## Introduction

Prothrombin complex concentrate (PCC) has been suggested as a measure to terminate trauma and dabigatran-induced bleeding. Owing to the conflicting data concerning such therapy, we investigated the impact of a four-factor PCC to terminate massive bleeding following the infliction of multiple trauma in dabigatran anticoagulated pigs.

## Methods

After ethical approval, 24 male pigs were administered dabigatran etexilate (30 mg/kg bid p.o.) for 3 days. On day 4, dabigatran in anaesthetised animals was infused prior to injury to achieve supra- therapeutic levels. Twelve minutes after infliction of bilateral femur fractures and standardised blunt liver injury, animals randomly received PCC (25, 50 or 100 IU/kg; *n *= 6) or placebo (*n *= 6). Time-adjusted blood loss as primary endpoint (observation period 300 minutes) and a panel of coagulation variables were continually measured. Data were analysed by two-way ANOVA. Data are mean ± SEM.

## Results

Concentrations of dabigatran prior to infliction of trauma was comparable between groups (590 ± 40 ng/ml). Anticoagulation with dabigatran and trauma caused severe coagulopathy as shown by prolonged TEM variables (CT, CFT), PT and aPTT. Following PCC application these effects were partially reversed. Due to ongoing blood loss both PT and TEM variables prolonged over time in PCC 25 IU/kg substituted animals. Accordingly, no-PCC (38.5 ± 4.7 ml/minute) and PCC 25 IU/kg (22.6 ± 5.5 ml/minute) animals showed highest blood loss (*P *< 0.05 vs. PCC 50 IU/kg and PCC 100 IU/kg) with a mean survival time of 106 minutes (no-PCC animals) and 204 minutes for PCC 25 IU/kg animals, respectively. All animals of the PCC 50 IU/kg and PCC 100 IU/kg group survived. Blood loss in both groups was comparable (PCC 50 IU/ kg: 5.9 ± 0.2 ml/minute; PCC 100 IU/kg 6.0 ± 0.3 ml/minute).

## Conclusion

The use of high doses of PCC reversed the anticoagulant effects of dabigatran, which was associated with a significant reduction of blood loss. However, the results of this study show that sufficient concentrations of PCC are necessary to overcome thrombin inhibition.

